# Pharmacological characterization of hetrombopag, a novel orally active human thrombopoietin receptor agonist

**DOI:** 10.1111/jcmm.13809

**Published:** 2018-08-29

**Authors:** Chengying Xie, Huajun Zhao, Xubin Bao, Haoyu Fu, Liguang Lou

**Affiliations:** ^1^ Shanghai Institute of Materia Medica Chinese Academy of Sciences Shanghai China; ^2^ College of Pharmaceutical Sciences Zhejiang Chinese Medical University Hangzhou Zhejiang China

**Keywords:** eltrombopag, hetrombopag, hollow‐fibre assay, thrombopoietin, thrombopoietin receptor

## Abstract

Nonpeptide thrombopoietin receptor (TPOR/MPL) agonists, such as eltrombopag, have been used to treat thrombocytopenia of various aetiologies. Here, we investigated the pharmacological properties of hetrombopag, a new orally active small‐molecule TPOR agonist, in preclinical models. Hetrombopag specifically stimulated proliferation and/or differentiation of human TPOR‐expressing cells, including 32D‐MPL and human hematopoietic stem cells, with low nanomolar EC_50_ values through stimulation of STAT, PI3K and ERK signalling pathways. Notably, hetrombopag effectively up‐regulated G_1_‐phase–related proteins, including p‐RB, Cyclin D1 and CDK4/6, normalized progression of the cell cycle, and prevented apoptosis by modulating BCL‐XL/BAK expression in 32D‐MPL cells. Moreover, hetrombopag and TPO acted additively in stimulating TPOR‐dependent signalling, promoting cell viability, and preventing apoptosis. Orally administered hetrombopag specifically promoted the viability and growth of 32D‐MPL cells in hollow fibres implanted into nude mice with much higher potency than that of the well‐known TPOR agonist, eltrombopag, in association with activation of TPOR‐dependent signal transduction in vivo. Taken together, our findings indicate that, given its favourable pharmacological characteristics, hetrombopag may represent a new, orally active, small‐molecule TPOR agonist for patients with thrombocytopenia.

## 1 INTRODUCTION

Thrombocytopenia, characterized by abnormally low platelet counts in circulating blood, is caused by decreased platelet production and/or accelerated platelet destruction. Thrombocytopenia is a frequent problem in a variety of medical conditions, including idiopathic thrombocytopenic purpura,^1^ myelodysplastic syndrome,^2^ chronic liver disease and acquired immunodeficiency syndrome.^3^ Chemotherapy and radiotherapy used in the treatment of cancer also frequently induce thrombocytopenia, which may limit further therapy.^4^ The main concept in treating thrombocytopenia is to eliminate the cause of the accelerated platelet destruction or increase platelet counts by stimulating the production of new platelets.

Platelet production originates from megakaryocyte precursor cells in the bone marrow. Thrombopoietin (TPO), a hematopoietic cytokine that is critically involved in regulating megakaryopoiesis,^5^, ^6^ exerts its biological function through activation of the thrombopoietin receptor (TPOR), also known as MPL (myeloproliferative leucemia virus oncogene), a type I transmembrane receptor expressed on hematopoietic stem cells, megakaryocytes and platelets.^7^ The interaction of TPO with its receptor induces transphosphorylation and activation of two Janus kinases, JAK2 and TYK2, which in turn results in the activation of signal transducer and activator of transcription 5 (STAT5), phosphoinositide‐3 kinase (PI3K) and mitogen‐activated protein kinase (MAPK),^8^, ^9^, ^10^ thereby stimulating megakaryocyte duplication and differentiation into platelets. Genetic elimination of TPO or TPOR in mice reduces the number of bone marrow megakaryocytes and circulating platelets by 80%‐90%,^11^, ^12^ suggesting that the TPO/TPOR signalling pathway is critical for maintaining the normal number of platelets in vivo. Thus, TPOR is recognized as an ideal target for the treatment of thrombocytopenia.

First‐generation TPOR agonists, such as recombinant human TPO (rhTPO) and PEGylated recombinant megakaryocyte growth and development factor (PEG‐rHuMGDF), have demonstrated efficacy in increasing platelet counts in the clinic.^5^ However, patients treated with these agonists often develop autoantibodies that cross‐react with and neutralize endogenous TPO, subsequently leading to severe thrombocytopenia or pancytopenia.^5^, ^13^ Thus, there is a need for drugs that are not structurally similar to TPO, and thus avoid immunogenic responses against the endogenous TPO, but are able to stimulate TPOR.^13^, ^14^ Second‐generation agents include peptide agents and nonpeptide compounds. To date, two different thrombopoietic drugs—romiplostim, a peptide compound, and eltrombopag, a nonpeptide TPOR agonist—have been approved for clinical use.^14^, ^15^, ^16^ Potential advantages of small‐molecule mimetics include their putative lack of immunogenicity, nonparenteral route of administration, as well as their lower costs of production. However, it has been reported that eltrombopag requires high daily dosing because of its low activity; this drug also comes with a black box warning for potential hepatotoxicity, with elevated liver enzyme levels or clinical signs of liver damage.^17^, ^18^, ^19^ These observations highlight the desirability of developing small‐molecule TPOR agonists with improved efficacy and reduced toxicity.

**Figure 1:**
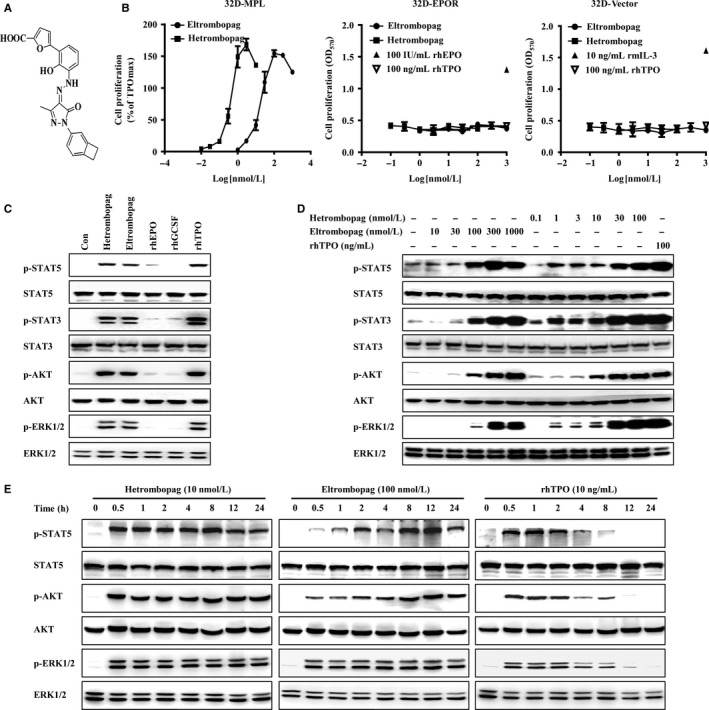
Hetrombopag specifically stimulates TPOR signalling in 32‐MPL cells and promotes their proliferation. A, The molecular structure of hetrombopag. B, Effects of hetrombopag and eltrombopag on the proliferation of 32D‐MPL (TPOR‐positive), 32D‐EPOR (EPOR‐positive) and 32D‐Vector (TPOR‐negative) cell lines. Recombinant mouse IL‐3 (rmIL‐3), recombinant human TPO (rhTPO) and recombinant human erythropoietin (rhEPO) were used as controls. Results were representative of at least three independent experiments and were expressed as means ± SD. C, Effects of different stimuli on TPOR‐dependent signal transduction in 32D‐MPL cells. 32D‐MPL cells were treated with 30 nmol/L hetrombopag, 1000 nmol/L eltrombopag, 100 ng/mL rhTPO, 20 ng/mL recombinant human granulocyte colony‐stimulating factor (rhGCSF), or 100 IU/mL rhEPO for 30 minutes. D, 32D‐MPL cells were treated with increasing concentrations of hetrombopag, eltrombopag or rhTPO (100 ng/mL) for 30 minutes. E, 32D‐MPL cells were treated with hetrombopag (10 nmol/L), eltrombopag (100 nmol/L) or rhTPO (10 ng/mL) for the indicated time. Whole‐cell lysates were analysed by Western blotting using the indicated antibodies

Here, employing proliferation assays using human TPOR‐transfected murine 32D cells (32D‐MPL), we demonstrated that hetrombopag (Figure 1A) is a novel, active, small‐molecule TPOR agonist. Compared with eltrombopag, hetrombopag, which is currently in Phase 3 clinical trials in China, more potently stimulated TPOR‐dependent signal transduction, promoted cell viability and/or differentiation, and prevented apoptosis in TPOR‐positive cells. Importantly, hetrombopag specifically enhanced the viability and promoted the growth of 32D‐MPL cells in hollow fibres implanted in nude mice, exhibiting much higher potency than eltrombopag in vivo.

## 2 MATERIALS AND METHODS

### 2.1 Materials

Hetrombopag, provided by Jiangsu Hengrui Medicine Co. Ltd. (Jiangsu, China), was prepared as 10 mmol/L stock solutions in dimethylsulphoxide for in vitro studies or in 0.5% carboxymethylcellulose containing 0.1% Tween‐80 for in vivo studies. Eltrombopag was purchased from Selleckchem (Houston, TX).

Recombinant human TPO (rhTPO), recombinant human erythropoietin (rhEPO), recombinant human granulocyte colony‐stimulating factor (rhGCSF), recombinant human stem cell factor (rhSCF) and recombinant mouse interleukin‐3 (rmIL‐3) were purchased from R&D Systems (Minneapolis, MN). ABT‐737 was obtained from Dalian Meilun Biotechnology Co. Ltd. (Jiangsu, China). Primary antibodies to p‐STAT5 (Y925), STAT5, p‐STAT3 (Y705), STAT3, p‐AKT, AKT, p‐ERK1/2, BCL‐XL, MCL‐1, BAK, p‐RB, RB, Cyclin D1, CDK4 and CDK6 were purchased from Cell Signaling Technology (Beverly, MA). Antibodies to ERK1/2 and β‐tubulin were purchased from Santa Cruz Biotechnology (Santa Cruz, CA, USA). Fluorescein isothiocyanate (FITC)‐conjugated anti‐CD41a (gpIIb), Phycoerythrin (PE)‐conjugated anti‐CD42a and isotype control antibodies were purchased from Thermo‐Fisher Scientific (Waltham, MA). Horseradish peroxidase (HRP)‐conjugated secondary anti‐rabbit/mouse IgG antibodies were purchased from Calbiochem (Millipore, Bedford, MA).

### 2.2 Cell culture

The murine myeloid 32D cell line was obtained from American Type Culture Collection (Manassas, VA). Human full‐length TPOR and EPOR cDNAs derived from MO7e and UT‐7 cells, respectively, were subcloned into pcDNA3.1 vector. Parental 32D cells were stably transfected with human TPOR (32D‐MPL), human EPOR (32D‐EPOR) or pcDNA3.1 vector (32D‐Vector) using Lipofectamine 2000 transfection reagent (Thermo‐Fisher Scientific). All cell lines were maintained in RPMI‐1640 medium supplemented with 10% foetal bovine serum (FBS) and 10% WEHI‐conditioned medium at 37°C in a humidified 5% CO_2_ atmosphere.

Human umbilical cord blood (CB) samples were obtained from normal full‐term deliveries according to institutional guidelines after obtaining informed consent. Human CB mononuclear cells (MNCs) were separated by Lympholyte‐H (Cedarlane, Burlington, Ontario, Canada) density gradient centrifugation. Human CB CD34^+^ cells were isolated using anti‐CD34 antibody‐conjugated magnetic beads (MACS; Miltenyi, Auburn, CA) according to the manufacturer's instructions.

### 2.3 Cell proliferation assay

Cytokine‐starved 32D‐MPL, 32D‐EPOR and 32D‐Vector cell lines (2 × 10^4^ cells/mL) were incubated in 96‐well plates with different concentrations of compounds for 3 days. CD34^+^ cells (2 × 10^4^ cells/mL) purified from normal human CB were incubated in Iscove's Modified Dulbecco's Medium (IMDM) containing 20% FBS supplemented with 100 ng/mL rhSCF, and incubated with different concentrations of compounds for 7 days. Cell proliferation was evaluated by 3‐(4,5‐dimethylthiazol‐2‐yl)‐2,5‐diphenyl tetrazolium bromide (MTT) assay. GraphPad Prism version 5 (GraphPad Software, San Diego, CA) curve‐fitting software was used to calculate half‐maximal effective concentration (EC_50_) values.

### 2.4 Western blotting

Cells were cytokine‐starved overnight and treated with different concentrations of compounds. After treatment, cells were washed twice with ice‐cold phosphate‐buffered saline and lysed in sodium dodecyl sulphate (SDS) sample buffer (100 mmol/L Tris‐HCl pH 6.8, 2% SDS, 20% glycerol, 1 mmol/L DTT). Cell lysates containing equal amounts of protein were separated by SDS‐PAGE and transferred to polyvinylidine difluoride membranes (Millipore). Blots were probed with specific primary antibodies and then with HRP‐conjugated secondary antibodies. Proteins were detected by immunoblotting using a Western blot imaging system (Clinx Science Instruments, Shanghai, China).

### 2.5 Flow cytometry analysis

Flow cytometry analyses were used to investigate the cell cycle and apoptosis, as described previously.^20^ Cytokine‐starved 32D‐MPL cells were treated with rhTPO, or compounds for 24 hours, after which cells were harvested and fixed in ice‐cold 70% ethanol at −20°C overnight. Fixed cells were then treated with 50 μg/mL of propidium iodide and RNase A at 37°C for 30 minutes. The cell‐cycle distribution was measured using a FACScan flow cytometer (BD Biosciences, San Jose, CA) and analysed with ModFit LT Mac V3.0 software. After treatment of 32D‐MPL cells with rhTPO or compounds for 72 hours, apoptosis was measured using a FITC Annexin V Apoptosis Detection Kit (BD Biosciences) according to the manufacturer's instructions. Fluorescence was acquired with an Accuri C6 Plus instrument (BD Biosciences).

### 2.6 Assay for colony forming unit‐megakaryocyte (CFU‐MK)

Colony assays for quantifying human megakaryocytic progenitors were performed using the MegaCult‐C kit (StemCell Technologies, Vancouver, Canada) according to the manufacturer's instructions. Human CD34^+^ cells were suspended in a double‐chamber slide (5.0 × 10^3^ cells/well), with hetrombopag, eltrombopag or rhTPO. After incubation for 10 days at 37°C in a humidified chamber with 5% CO_2_, CFU‐MK was detected by staining the cells with anti‐human CD41 antibody. The number of CFU‐MK was counted and subdivided by colony size, and classified as small (3‐20 cells/colony), medium (21‐49 cells/colony) and large (50 cells/colony).

### 2.7 Cell differentiation assay

Human CD34^+^ cells were plated in IMDM containing 20% FBS and 100 ng/mL rhSCF and then treated with rhTPO or compounds for 10 days. Cells were stained with anti‐human CD41‐FITC, isotype control antibody, or PBS (autofluorescence control). Flow cytometric analyses were performed using a FACScan flow cytometer (BD Biosciences). Gates for CD41^+^ cells were based on the negative control (ie, SCF‐treated cultures). Data were presented as a percentage, calculated as follows: TPO_max_ = (% CD41_sample_ − % CD41_SCF_)/(% CD41_TPO_ − % CD41_SCF_). Cells which were stained with both anti‐human CD41‐FITC and CD42a‐PE represented mature MK (CD41^+^/CD42a^+^ cells).

### 2.8 Megakaryocyte proplatelet formation

The megakaryocyte proplatelet formation was conducted as described previously.^21^ Human CB CD34^+^ cells were cultured with hetrombopag, eltrombopag or rhTPO to stimulate MK differentiation. At day 14, the proplatelet‐bearing MK were counted using a Leica DMIRB inverted light microscope (Leica Microsystems, Australia). In addition, the number of CD41^+^ cells was determined by flow cytometry. The number of proplatelet‐bearing MK was determined as (proplatelet‐bearing MK/total CD41^+^cells)*1000.

### 2.9 Hollow‐fibre assay

All animal experiments were approved by the Institute Animal Review Board of Shanghai Institute of Materia Medica, Chinese Academy of Sciences, with firm adherence to ethical guidelines for the care and use of animals.

In vivo hollow‐fibre assays were performed as described previously.^22^ Briefly, a hollow fibre was filled with the cell suspension (1 × 10^7^ cells/mL) and subsequently sealed in 2‐cm segments. The hollow‐fibre capsules were inserted into subcutaneous tissue through a 10‐gauge trocar, after which mice were randomly assigned to control and treatment groups. Control groups were given vehicle alone, and treatment groups received oral hetrombopag or eltrombopag. Mice were sacrificed at the end of treatment, and the subcutaneously implanted cells were collected in culture medium and subjected to MTT assays.

Pharmacokinetics/pharmacodynamics studies were carried out as described previously.^23^ Mice implanted with 32D‐MPL cell‐containing hollow fibres received a single dose of 18 mg/kg hetrombopag or vehicle; 32D‐MPL hollow fibres and blood were collected at different times postdosing. Concentrations of hetrombopag in plasma were determined by HPLC/tandem mass spectrometry. Cells in hollow fibres were subjected to MTT assay or Western blot analysis.

### 2.10 Statistics

Data are presented as means ± SD (in vitro) or means ± SEM (in vivo), and significance was assessed with Student's *t* test. Differences were considered significant at *P *<* *0.05.

## 3 RESULTS

### 3.1 Hetrombopag is a nonpeptide agonist of human TPOR

Screening of a chemical compound series by proliferation assays using 32D cells stably transfected with human TPOR (32D‐MPL) led to our identification of the TPOR agonist hetrombopag (Figure 1A). The proliferation‐stimulating activity of this compound towards 32D‐MPL cells was calculated relative to the maximum proliferative activity of 100 ng/mL rhTPO. Hetrombopag and eltrombopag both induced a concentration‐dependent increase in the proliferation of 32D‐MPL cells, with EC_50_ values of 0.4 and 13.4 nmol/L, respectively (Figure 1B). In control experiments, hetrombopag, eltrombopag, as well as rhTPO had no effect on the proliferation of 32D‐EPOR cells, stably transfected with human EPOR, whereas rhEPO stimulated 32D‐EPOR cell proliferation. Also, none of these three agents affected the proliferation of TPOR‐negative 32D‐Vector cells.

Thrombopoietin (TPO) acts through binding to TPOR to stimulate multiple intracellular signalling pathways, including JAK/STAT, PI3K/AKT and ERK1/2.^8^, ^9^, ^10^ To elucidate the molecular mechanisms by which hetrombopag promoted proliferation, we examined whether hetrombopag activated these intracellular signalling pathways in 32D‐MPL cells. Control experiments confirmed that the cytokines rhGCSF or rhEPO had no effect on TPO/TPOR signalling‐dependent phosphorylation of these targets (Figure 1C). Similar to rhTPO, both hetrombopag and eltrombopag induced phosphorylation of the major components of TPO‐mediated signalling, including STAT3, STAT5, ERK1/2, and AKT (Figure 1C). Furthermore, hetrombopag stimulated the phosphorylation of these TPOR downstream effectors in a concentration‐dependent manner (Figure 1D). In keeping with its proliferation‐stimulating activity, hetrombopag exerted much stronger stimulation of TPO/TPOR signalling in 32D‐MPL cells than did eltrombopag (Figure 1D). Further study showed that TPOR downstream effectors peaked at 0.5‐2 hours, and decreased to normal level at 12 hours after treatment with rhTPO. However, hetrombopag treatment sustained high levels of signalling for significantly longer periods (0.5‐24 hours), and stimulated with eltrombopag, these signalling molecules became maximally active at later time‐points (2‐24 hours; Figure 1E). Taken together, these results indicate that hetrombopag stimulates intracellular TPO signalling pathways and promotes cell proliferation in a TPOR‐dependent manner.

### 3.2 Hetrombopag promotes proliferation and differentiation of human megakaryocyte progenitor cells

**Figure 2:**
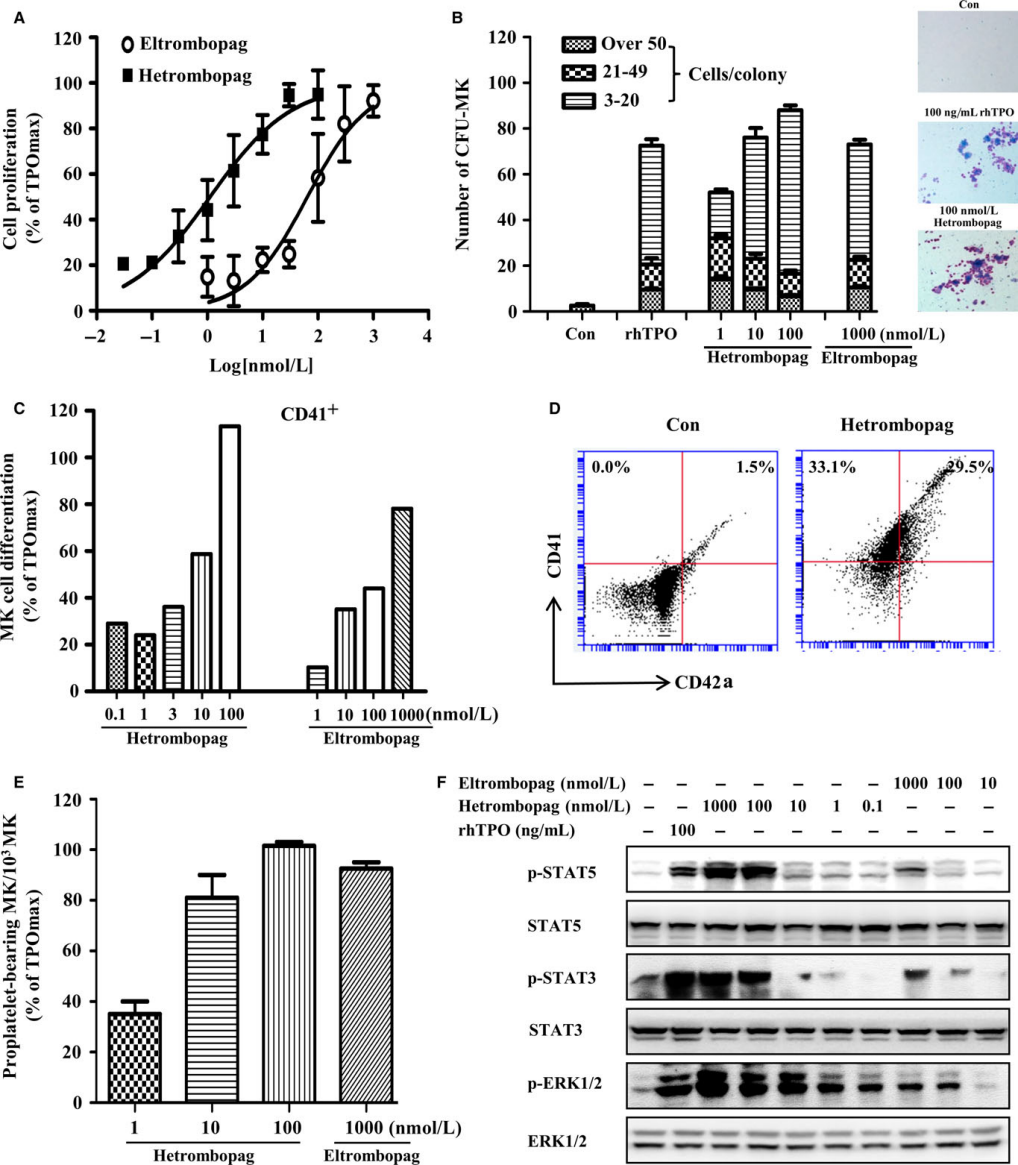
Hetrombopag promotes megaryopiesis and proplatelet production in vitro. A, Proliferation of human CB‐derived CD34^+^ cells induced by hetrombopag. Cell viability was determined by MTT assay. B, Megakaryocyte colony formation from human CB‐derived CD34^+^ cells induced by hetrombopag. The number of CFU‐MK was counted and subdivided by colony size, and classified as small (3‐20 cells/colony), medium (21‐49 cells/colony), and large (50 cells/colony). Immunohistochemical identification of typical human megakaryocyte colonies (right). C, Flow cytometry profile for the differentiation of human CB‐derived CD34^+^ cells induced by hetrombopag (CD41^+^). D, Flow cytometry profile of cultured MK (100 nmol/L hetrombopag), stained with anti‐human CD41‐FITC and CD42a‐PE. Dots in the upper right quadrant represented mature MK (CD41^+^/CD42a^+^ cells). E, Hetrombopag‐stimulated proplatelet‐bearing MK. The number of proplatelet‐bearing MK was determined as (proplatelet‐bearing MK/total CD41^+^ cells)*1000. F, Hetrombopag‐stimulated phosphorylation of STAT and ERK1/2 in megakaryocytes derived from human CD34^+^ cells. The proliferation‐ and differentiation‐stimulating activity of compounds was calculated relative to the maximum activity of 100 ng/mL rhTPO

Activation of TPOR expressed on the surface of megakaryocytes and their precursors trigger the expression of genes involved in the megakaryocytic pathway and lead to the release of platelets.^24^ Accordingly, we next investigated the effect of hetrombopag on megakaryopoiesis in TPOR‐positive human CB‐derived CD34^+^ cells, used as a source of hematopoietic stem cells. Both hetrombopag and eltrombopag stimulated the proliferation of human CB‐derived CD34^+^ cells, with EC_50_ values of 2.3 and 86.2 nmol/L, respectively (Figure 2A). In semisolid culture systems using the MegaCult‐C kit, hetrombopag, eltrombopag, as well as rhTPO, increased the number of CFU‐MK from human CB‐CD34^+^ hematopoietic progenitor cells. The activity of 100 nmol/L hetrombopag was comparable to that of 100 ng/mL rhTPO (Figure 2B). The total number of MK (CD41^+^) increased after treated with hetrombopag and eltrombopag in a concentration‐dependent manner, with EC_50_ values of 4.5 and 80.8 nmol/L, respectively (Figure 2C). The percentage of mature MK (CD41^+^/CD42a^+^) also increased after treatment with hetrombopag (Figure 2D). Proplatelet formation and platelet release could be a consequence of increased MK proliferation and maturation.^21^ As expected, hetrombopag stimulated MK proplatelet formation from human CB‐CD34^+^ cells in vitro (Figure 2E). Corresponding to its MK‐stimulating activity, hetrombopag induced a concentration‐dependent increase in the phosphorylation of STAT3, STAT5 and ERK1/2 in human CB CD34^+^ cells, exerting a much stronger effect than eltrombopag (Figure 2F). Taken together, these results suggest that hetrombopag is capable of stimulating the proliferation and differentiation of megakaryocyte progenitor cells, and then proplatelet production via TPOR signalling.

### 3.3 Hetrombopag normalizes cell‐cycle progression and prevents apoptosis in 32D‐MPL cells

**Figure 3:**
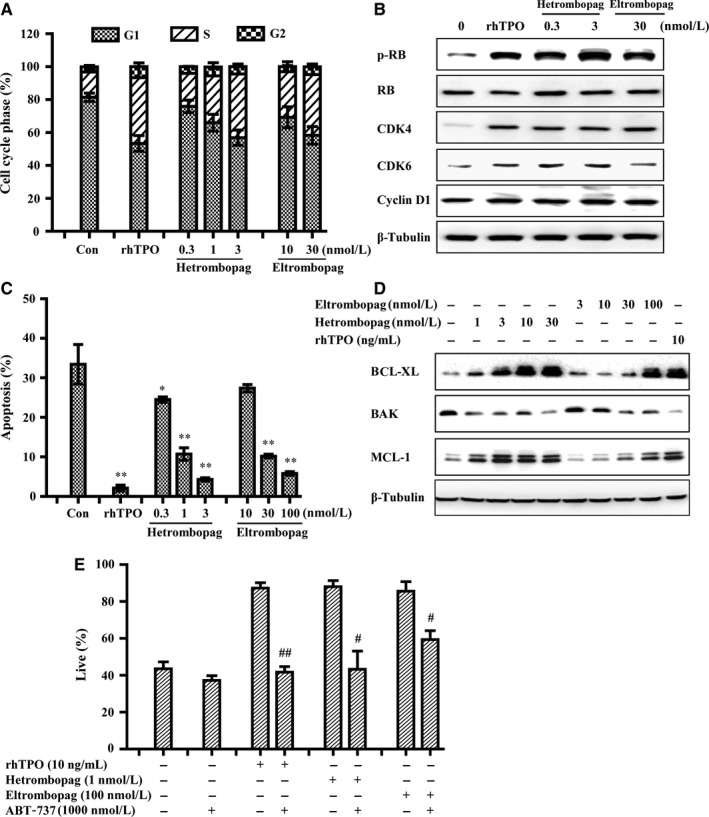
Hetrombopag normalizes cell‐cycle progression and prevents apoptosis in 32D‐MPL cells. A, Cell‐cycle phase histograms of 32D‐MPL cells following treatment with the indicated concentrations of hetrombopag or eltrombopag, or 10 ng/mL rhTPO, for 24 hours. B, After treatment of 32D‐MPL cells with hetrombopag, eltrombopag or 10 ng/mL rhTPO for 24 hours, whole‐cell lysates were immunoblotted with the indicated antibodies. C, 32D‐MPL cells were treated with the indicated concentrations of hetrombopag or eltrombopag, or 10 ng/mL rhTPO for 72 hours, and then analysed by annexin V‐FITC/PI staining and flow cytometry. D, After treatment of 32D‐MPL cells with hetrombopag, eltrombopag or rhTPO for 48 hours, whole‐cell lysates were immunoblotted with the indicated antibodies. E, 32D‐MPL cells were treated with hetrombopag, eltrombopag, rhTPO, ABT‐737 or the indicated combinations for 72 hours, and then analysed by annexin V‐FITC/PI staining and flow cytometry. *P < 0.05, **P <* *0.01 vs Con; ^#^ *P *<* *0.05, ^##^ *P *<* *0.01 versus treatment with either single agent

To explore the mechanism underlying hetrombopag promotion of cell viability, we further used 32D‐MPL cells to investigate the effects of hetrombopag on the cell‐cycle profile. Withdrawal of cytokines for 24 hours led to G_1_ cell arrest. Subsequent treatment with hetrombopag, eltrombopag, or rhTPO caused 32D‐MPL cells to re‐enter the cell cycle, increasing the proportion of cells in G_2_ and S phases and decreasing the proportion of cells in G_1_ phase (Figure 3A). Consistent with this, treatment with hetrombopag or eltrombopag reversed the decrease in G_1‐_phase–related proteins, including p‐RB, Cyclin D and CDK4/6, induced by cytokine withdrawal (Figure 3B).

A previous study showed that the TPOR agonist eltrombopag exerts antiapoptotic activity in TPOR‐positive cells.^25^ Prolonged withdrawal of cytokines (72 hours) induced apoptosis of 32D‐MPL cells, an effect that was reduced by rhTPO, hetrombopag, or eltrombopag (Figure 3C). Next, we further elucidated the possible molecular mechanisms underlying this processes. Changing protein expression levels of BCL‐2 family members, which are highly dynamically regulated, is a key mechanism for tipping the balance towards or away from apoptotic cell death.^26^, ^27^ Similar to rhTPO, both eltrombopag and hetrombopag increased expression of the antiapoptotic family members BCL‐XL and MCL‐1, and decreased expression of pro‐apoptotic BAK (Figure 3D). Furthermore, the antiapoptotic action of hetrombopag was prevented by ABT‐737, a BCL‐2/BCL‐XL inhibitor (Figure 3E). These results demonstrate for the first time that TPOR agonists drive appropriate cell cycling of 32D‐MPL cells through upregulation of G_1_‐phase–related regulatory proteins, and prevent apoptosis through alterations in BCL‐XL/BAK expression.

### 3.4 Additive effect of hetrombopag in combination with rhTPO

**Figure 4:**
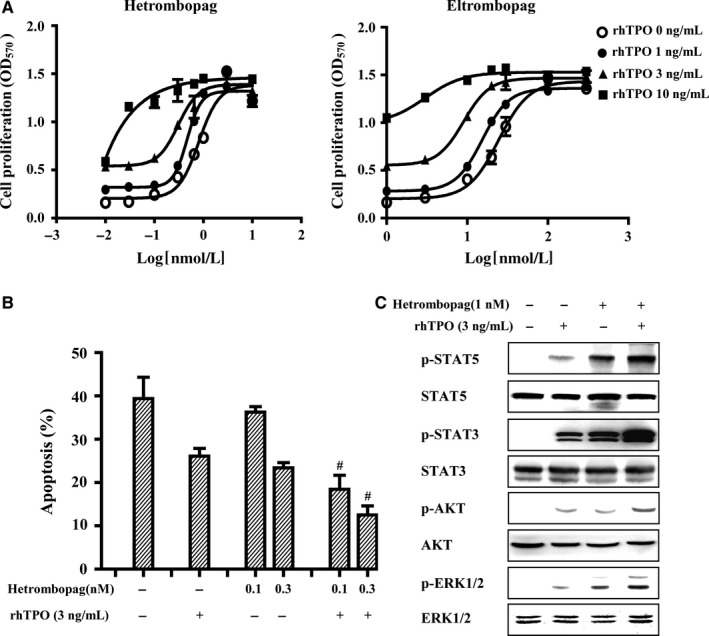
Combined treatment of 32D‐MPL cells with rhTPO and hetrombopag. A, 32D‐MPL cells were incubated with the indicated concentrations of hetrombopag or eltrombopag, alone or in combination with rhTPO, for 72 hours. Cell viability was determined by MTT assay. B, 32D‐MPL cells were incubated with hetrombopag, rhTPO, or their combination for 72 hours, and analysed by annexin V‐FITC/PI staining and flow cytometry. ^#^ *P* < 0.05 vs treatment with either single agent. C, 32D‐MPL cells were incubated with hetrombopag, rhTPO, or their combination for 30 minutes. Whole‐cell lysates were analysed by Western blotting using the indicated antibodies

A previous study showed that effects of eltrombopag and rhTPO in TPOR‐positive cell lines were additive.^25^ Accordingly, we investigated whether this was also the case for hetrombopag. As shown in Figure 4A, hetrombopag, such as eltrombopag, exerted an additive proliferation‐promoting effect in 32D‐MPL cells when combined with rhTPO. Moreover, the combination of hetrombopag and rhTPO exerted enhanced antiapoptotic effects in 32D‐MPL cells compared with either agent alone (Figure 4B). To gain insight into the mechanisms of this additivity, we analysed the effects of combined treatment with these two agents on cellular TPOR signalling. Hetrombopag combined with rhTPO stimulated the phosphorylation of STAT3, STAT5, AKT and ERK1/2 in 32D‐MPL cells, exerting augmented stimulatory effects on TPOR signalling compared with each single agent alone (Figure 4C). These results suggest that hetrombopag interacts specifically with TPOR and exerts an additive agonistic effect with rhTPO.

### 3.5 Analysis of the pharmacodynamics and pharmacokinetics of hetrombopag using hollow‐fibre assay

**Figure 5:**
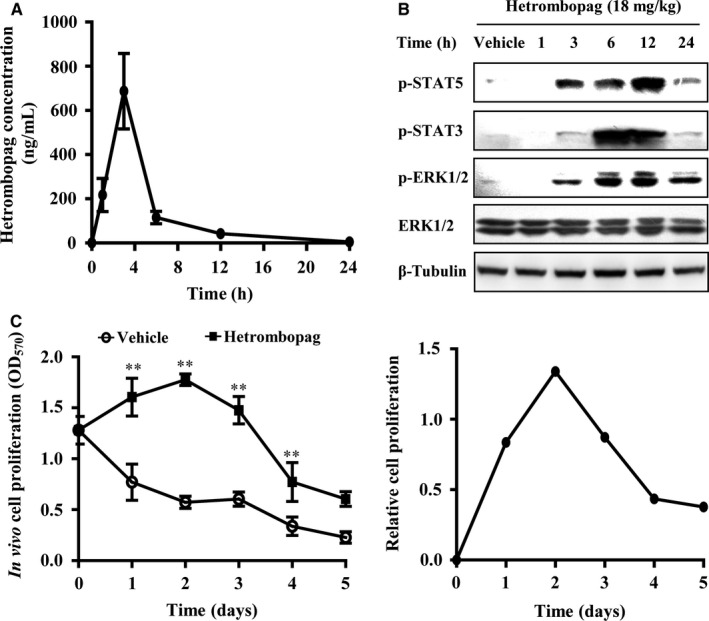
In vivo pharmacodynamic and pharmacokinetic analysis of hetrombopag by hollow‐fibre assay. A and B, Hollow fibres were filled with 32D‐MPL cells and then subcutaneously implanted into nude mice. Control groups were given orally administered vehicle, and treatment groups were given a single oral dose of 18 mg/kg hetrombopag. At the indicated times, whole blood was obtained for drug concentration analysis (A), and whole‐cell lysates of 32D‐MPL cells from hollow fibres were analysed by Western blotting (B). C, In vivo efficacy of a single oral dose of hetrombopag. Mice implanted with 32D‐MPL hollow fibres were orally administered a single 18‐mg/kg dose of hetrombopag. At the indicated times, mice were sacrificed and cells from the fibres were collected. Proliferation of 32D‐MPL cells was determined by MTT assay. Relative cell proliferation was calculated as (OD_hetrombopag_ − OD_vehicle_). Data are presented as means ± SEM (n = 6; **P <* *0.01 vs vehicle)

Our previous study showed that hollow‐fibre assay using 32D‐MPL cells is a specific and efficient approach for rapidly evaluating the in vivo activity of small‐molecule TPOR agonists.^22^ Here, we used this assay to evaluate the pharmacodynamics of hetrombopag on the TPOR‐dependent signalling pathway and its relationship to the pharmacokinetics of hetrombopag. After oral administration of a single 18‐mg/kg dose of hetrombopag, the plasma concentration of hetrombopag peaked at 687 ± 342 ng/mL at 3 hours and decreased to 4.5 ± 0.5 ng/mL at 24 hours (Figure 5A). In association with these changes, the phosphorylation of STAT3, STAT5 and ERK1/2 was first detected at 3 hours, peaked at 6‐12 hours, and lasted about 24 hours (Figure 5B).

We next examined the effect of administration of a single oral 18‐mg/kg dose of hetrombopag on cell viability over the course of 5 days. These experiments showed that the number of live cells in hollow fibres in the vehicle groups decreased in a time‐dependent manner. However, the number of 32D‐MPL cells in hollow fibres changed dynamically after a single oral dose of hetrombopag (18 mg/kg), reaching peak optical density (OD) at 2 days and decreasing from days 3 to 5 (Figure 5C). Thus, hetrombopag showed oral bioavailability, stimulated proliferation, and prevented apoptosis of 32D‐MPL cells in vivo through TPOR‐dependent signalling.

### 3.6 In vivo activity of hetrombopag in hollow‐fibre assay

**Figure 6:**
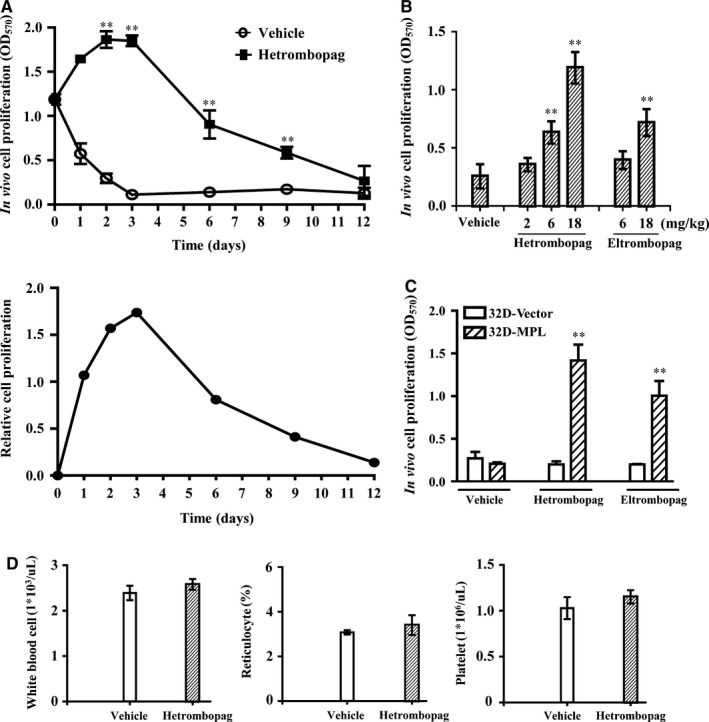
In vivo activity of hetrombopag, determined by hollow‐fibre assay. A, Schedule‐dependent efficacy of hetrombopag. Mice implanted with 32D‐MPL hollow fibres were given an 18‐mg/kg oral dose of hetrombopag daily for the indicated duration. Relative cell proliferation was calculated as (OD_hetrombopag_ − OD_vehicle_). B, Dose‐dependent efficacy of hetrombopag. Mice implanted with 32D‐MPL hollow fibres were given the indicated oral dose of hetrombopag or eltrombopag daily for 3 days. C, The specificity of hetrombopag in the in vivo model. Mice implanted with 32D‐MPL or 32D‐Vector hollow fibres were given an oral dose of hetrombopag (18 mg/kg) or eltrombopag (40 mg/kg) daily for 3 days. D, Mice implanted with 32D‐MPL hollow fibres were given an oral dose of hetrombopag (18 mg/kg) daily for 3 days. At the end of treatment, peripheral blood cells were collected and counted using a Sysmex haematology analyzer. Proliferation of cells obtained from the fibres was determined by MTT assay. Data are presented as means ± SEM (n = 6; **P <* *0.01 vs vehicle)

On the basis of pharmacodynamic/pharmacokinetic studies of hetrombopag using hollow‐fibre assay, we used a once‐daily dosing regimen in nude mice subcutaneously implanted with hollow fibres containing 32D‐MPL cells to investigate the efficacy of hetrombopag in vivo. Daily oral administration of 18 mg/kg hetrombopag for 12 days significantly stimulated proliferation and prevented apoptosis of 32D‐MPL cells in hollow fibres in a time‐dependent manner (Figure 6A). The number of 32D‐MPL cells reached a maximum after 3 days of daily oral administration of hetrombopag, and then decreased within 12 days. In contrast, few live cells were present in hollow fibres in the vehicle group after 3 days of treatment (Figure 6A). These results suggest that daily oral dosing for 3 consecutive days is an optimal schedule. Furthermore, consistent with in vitro results, daily oral administration of hetrombopag for 3 consecutive days showed greater efficacy in stimulating the in vivo proliferation of 32D‐MPL cells than eltrombopag (Figure 6B). The in vivo efficacy of hetrombopag was also dose‐dependent, and was significant at doses of 6 mg/kg or greater (*P* < 0.01).

Further study showed that neither hetrombopag nor eltrombopag had an effect on the in vivo proliferation of TPOR‐negative 32D‐Vector cells in hollow fibres (Figure 6C). Furthermore, hetrombopag had no effect on the counts of white blood cells, reticulocytes, or platelets in the peripheral blood of mice (Figure 6D). Taken together, the in vivo results obtained by hollow‐fibre assay indicate that orally administered hetrombopag specifically activates human TPOR signalling and is more efficacious than eltrombopag.

## 4 DISCUSSION

The TPO/TPOR signalling pathway plays a central role in regulating both megakaryopoiesis and platelet production. Thus, the use of TPOR agonists represents a major approach for the treatment of thrombocytopenia.^28^ Among these agonists, nonpeptide agonists of TPOR, such as eltrombopag, butyzamide and NIP‐004, have advantages over peptide agonists in that they are orally bioavailable and are unlikely to induce an immune response.^25^, ^29^, ^30^ Here, we identified hetrombopag, a member of the biarylhydrazone class of compounds, as a novel, orally active TPOR agonist. Hetrombopag was capable of activating TPO‐specific signal transduction, stimulating proliferation and differentiation, and preventing apoptosis in TPOR‐positive cells. Importantly, hetrombopag exhibited greater efficacy than eltrombopag both in vitro and in vivo, and exerted effects that were additive with those of rhTPO.

TPOR belongs to the type I cytokine receptor family, which includes EPOR and GCSF receptors. Like EPOR and GCSF receptors, this homodimeric receptor undergoes a major conformational change upon agonist binding, followed by phosphorylation of its intracellular domain and various secondary signalling molecules.^6^ Although numerous growth factors had been shown to stimulate platelet production, the extent to which platelet count increases is of marginal clinical significance.^31^ Our study showed that neither rhGCSF nor rhEPO had an effect on TPOR‐activated signalling or proliferation in TPOR‐positive 32D‐MPL cells. However, hetrombopag and eltrombopag, as well as rhTPO, specifically stimulated phosphorylation of STAT3, STAT5, AKT and ERK1/2, and promoted the proliferation of 32D‐MPL cells. Hetrombopag exhibited high activity with EC_50_ values in the picomolar range, a potency 30‐times greater activity than that of eltrombopag. The activity of hetrombopag was dependent on TPOR, as evidenced by the fact that it did not support the proliferation of cell lines stably transfected with 32D‐Vector or 32D‐EPOR, which do not express TPOR, but do express the IL‐3 receptor and EPOR, respectively. Consistent with a previous study demonstrating that TPOR agonists stimulate megakaryopoiesis and hence lead to platelet production,^25^, ^29^ hetrombopag was capable of stimulating the proliferation of CD34^+^ hematopoietic progenitor cells and promoting their differentiation into megakaryocytes and then proplatelet production, which involved the stimulation of TPOR signalling.

TPO/TPOR signalling is able to inactivate or induce more than 100 proteins, and generally exerts a prosurvival effect.^32^ For the first time, we found that TPOR agonists, including hetrombopag and eltrombopag, as well as rhTPO, effectively up‐regulated the G_1_‐phase–related proteins p‐RB, Cyclin D1 and CDK4/6, and normalized the cell‐cycle profile in 32D‐MPL cells. To the best of our knowledge, these interesting effects on cell cycling revealed by our investigation of hetrombopag have not been investigated in previous studies on TPOR agonists. A previous study showed that TPOR agonists affect the apoptotic profile of platelets in patients with chronic immune thrombocytopenia.^32^ TPOR agonists have also been reported to exert antiapoptotic activity in TPOR‐positive cells.^25^ Here, hetrombopag suppressed caspase and poly‐(ADP‐ribose) polymerase (PARP) cleavage (data not shown) and prevented apoptosis of 32D‐MPL cells. In addition, hetrombopag‐stimulated AKT signalling, which has been shown to inhibit apoptosis through regulation of BCL‐2 family proteins.^33^ Consistent with these reports, we found that hetrombopag‐stimulated BCL‐XL and MCL‐1 expression, and inhibited BAK expression. Furthermore, the antiapoptotic effect of hetrombopag was prevented by the BCL‐2/BCL‐XL inhibitor ABT‐737, confirming that hetrombopag attenuates apoptosis through modulation of BCL‐XL/BAK.

Romiplostim is thought to bind directly to the TPO‐binding site on TPOR, whereas eltrombopag has been proposed to bind to TPOR near its membrane insertion site, away from the active site.^34^ Hetrombopag, similar to eltrombopag, did not bind to the same site on TPOR as TPO, which prevented competitive binding and allowed hetrombopag and TPO to exert additive cell‐signalling effects. Their combination also produced additive prosurvival and antiapoptotic activities in TPOR‐positive cells, suggesting that hetrombopag and endogenous TPO could act in concert.

Notably, published animal data on nonpeptide agonists such as eltrombopag are often limited because such agents are only active in humans and chimpanzees.^25^, ^29^, ^30^ The hollow‐fibre assay is currently in routine use as a screening tool for anticancer drug discovery.^35^ Our previous study showed that the 32D‐MPL hollow‐fibre assay was a specific and efficient model for rapidly evaluating the in vivo activity of small‐molecule TPOR agonists.^22^ Here, this assay was used to investigate the pharmacokinetic and pharmacodynamic characteristics of hetrombopag. Preliminary results from this assay suggested that hetrombopag was orally available, showing peak plasma concentrations 3 hours after administration of a single 18‐mg/kg dose and rapidly decreasing within 24 hours; by comparison, TPOR‐dependent signalling peaked at 6‐12 hours and lasted for at least 24 hours. The discrepancy between time profiles of plasma concentration and TPOR‐dependent signalling could reflect the distribution of hetrombopag or the existence of double‐peaks in plasma in hetrombopag concentration‐time profiles, as reported in a clinical study.^36^ However, double‐peaks of plasma concentrations were not observed at the time‐points used in our experimental paradigm. Our pharmacodynamic and pharmacokinetic data for hetrombopag based on hollow‐fibre assays indicated that a once‐daily dosing regimen would be appropriate for clinical use, a finding that is generally consistent with the results of a Phase 1 study.^36^

In vivo hollow‐fibre assay results indicated that oral doses of hetrombopag of 6 mg/kg and above were effective, as measured by stimulation of TPOR signal transduction and proliferation of TPOR‐expressing cells. Although we could not confirm human platelet production using this in vivo assay, the efficacy of hetrombopag has also been demonstrated in the clinic. In a Phase 1 study, once‐daily oral administration of 10 mg hetrombopag to healthy volunteers for 14 days increased the number of platelets, resulting in more than a 50% increase over baseline platelet counts.^36^ A dose range of 2.5‐7.5 mg once‐daily could be considered a recommended regimen for patients with chronic immune thrombocytopenia. The pharmacological data obtained using the in vivo hollow‐fibre model demonstrate that this assay is suitable for preliminary predictions of dose‐response relationships of orally active TPOR agonists in the clinic. The strong stimulatory activity and good pharmacokinetic profile of TPOR ensure improved clinical efficacy of hetrombopag compared with eltrombopag. Low doses of hetrombopag that achieve a comparable pharmacological effect might also reduce the potential for off‐target toxicity. In a previous eltrombopag study, aminotransferase was found to be elevated by 2%‐4%, and treatment was interrupted in several subjects owing to liver toxicity,^18^ but no preclinically or clinically significant abnormalities in hepatobiliary laboratory tests were found.^36^

In conclusion, we identified the nonpeptide, hetrombopag, as a TPOR agonist, and showed that it mimicked, and was additive with, the biological activity of TPO. Moreover, hetrombopag exhibited greater pharmacological efficacy than eltrombopag both in vitro and in vivo. These preclinical results provide the foundation for clinical evaluations of hetrombopag for treating thrombocytopenia of various aetiologies.

## CONFLICT OF INTEREST

The authors declare no conflict of interest.
